# Lung Clear “Sugar” Cell Tumor and JAK V617F Positive Essential Thrombocythemia: a Simple Coincidence?

**DOI:** 10.4084/MJHID.2013.021

**Published:** 2013-04-10

**Authors:** Volkan Yazak, Gokhan Sargin, Irfan Yavasoglu, Gurhan Kadikoylu, Canten Tataroglu, Gokay Bozkurt, Zahit Bolaman

**Affiliations:** 1 Adnan Menderes University Medical Faculty, Division of Hematology, Aydin, Turkey; 2 Adnan Menderes University Medical Faculty, Division of Pathology, Aydin, Turkey; 3 Adnan Menderes University Medical Faculty, Division of Medical Genetics, Aydin, Turkey

## Abstract

The primary clear cell tumor of the lung is an extremely rare benign tumor. This tumor is called “sugar tumor” since clear cell tumor of the lung contains abundant glycogen. We here present a case of lung clear cell tumor of the lung associated to essential thrombocythemia. To the best of our knowledge, there is no report about this association.

A 44-Year-Old Woman admitted to our clinic with a 2-month history of fatigue. On physical examination, the spleen was 3 cm palpable below the left costal margin on the mid axillary line. The laboratory tests revealed an elevated platelet counts (1,014,000/mm^3^). A pulmonary nodule (3,5 cm) was detected in the upper right lobe on the chest X-ray. Then, thoracic computed tomography (CT) was planned. The nodule looked like benign pattern on CT scan and total excision was performed for curative and diagnostic treatment.

Microscopically, the tumor was composed of nests of rounded or oval cells with distinct cell borders, optically clear cytoplasm and small nucleus. By immunohistochemistry, tumor cells were positive for HMB-45, NSE and focal S100 antigen. And, it was diagnosed as clear “sugar” cell tumor. After tumor excision the lasting thrombocytosis induced us to perform bone marrow biopsy and JAK2 mutation research.

Diagnosis of Essential Thrombocythemia was made. In conclusion, it is important to make an evaluation for myeloproliferative diseases in clear “sugar” cell tumor in adults if thrombocytosis was lasting after treatment.

## Introduction

“Sugar tumor” was first described by Castleman and Liebow in 1963.^1^ The primary clear cell tumor of the lung is an extremely rare benign tumor. Tumor cells contain abundant glycogen. It may located under the pleura in any lobe and have no communication with bronchus. The tumor is usually accidentally detected on X-rays as a circumscribed peripheral nodule. The lesion appears in the middle-aged and elderly patients with no clinical symptoms. Cellular origin of the tumor still remains enigmatic. It is thought that tumor cells might be derived from perivascular epithelioid cells (PECs) and described as PEComas by several authors.^2^

Thrombocytosis can be found in patient with different neoplasms^3^ furthermore second malignancies may occur in essential thrombocythemia.^4^

Here we report a case of lung clear cell tumor presenting contemporary essential thrombocythemia. To the best of our knowledge, there is no report about this association.

## Case Report

A 44-year-old woman was admitted to our clinic with a 2-month history of fatigue, cough, dyspnea and fever with hemoptysis. There was not familiarity for lung tumor or tuberculosis. On pathological examination, the spleen was 3 cm palpable below the left costal margin on the mid axillary line. The physical examination of other organs was normal. The results of laboratory tests showed hemoglobin level of 12, 2 g/dl, hematocrit 37,5%, white blood cell count 10,200/mm^3^, platelet count 1,014,000/mm^3^. The peripheral blood smear demonstrated normochromic normocytic red blood cells, an increased platelet number, a normal differential leucocyte count with 70% neutrophil, 2% eosinophil and 28% lymphocyte.

The laboratory parameters were as follows: Iron (Fe): 49 ug/dl, transferrin: 210 ug/dl, Fe saturation: 16%, UIBC: 252 ug/dl, TIBC: 301 ug/dl, VitB12: 228 pg/ml, and ferritin: 88 ng/ml. Urine analysis revealed normal findings. Sedimentation rate was 18 mm/h; C-reactive protein was 5 ng/ml.

**Figure 1:**
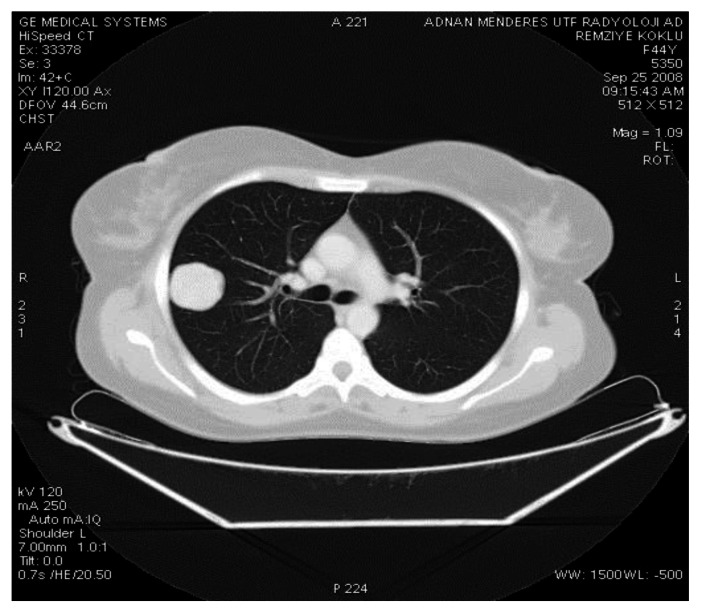
In CT parenchyma window in right lung upper lobe apical segment, a solid peripheral subpleural situated nodule which is 37 mm in diameter and has a homogeneous formation showing umbilication in lobule with a contour-good bordered medial part.

A pulmonary nodule (3,5 cm) was detected in the upper right lobe on the chest X-ray. Then, thoracic CT was planned. Thoracic CT revealed a solid peripheral subpleural nodule in the right upper lung lobe apical segment with diameter of 37 mm. Nodule has homogeneous pattern showing umbilication in lobule contour-good bordered medial part (Figure 1).

Abdominal CT scan, performed to exclude metastatic renal cell carcinoma, showed only mild splenomegaly. The nodule looked like benign pattern on CT scan and total excision was performed for curative and diagnostic treatment. Microscopically, the tumor was composed of nests of rounded or oval cells with distinct cell borders, optically clear cytoplasm and small nuclei. By immunohistochemistry, tumor cells were positive for HMB-45, NSE and focal S100 antigen. The immunohistochemical staining for synaptophysin was negative. And, it was diagnosed as clear “sugar” cell tumor.

Malignant features of Clear “Sugar” Cell Tumor were excluded by imaging investigations before and after surgical tumor treatment.

However, considering that thrombocytosis continued after the surgical removal of the clear cell tumor in the third month in absence of infections, bone marrow biopsy was performed. The cellularity was 70% and the myeloid/erythroid series ratio was 4. Megakaryocytic series was hyperplasic. There were no atypical cells. Iron stores were normal. Normal karyotype was provided by GTL banding (G banding). JAK V617F heterozygote mutation was detected by polymerase chain reaction (PCR). Essential thrombocythemia was diagnosed according to the World Health Organization (WHO) criteria.^5^

Being the platelet count over 1,000,000/mm^3^ in the 3^rd^ month after the surgical intervention, the patient was started on therapy with oncocarbide. At present she is at 18^th^ month of the follow-up in good condition with a normal abdominal and thoracic CT.

## Discussion

After the excision of lung clear cell tumor, our patient continued to present thrombocytosis and splenomegaly, without reasons of reactive thrombocytosis, the bone marrow picture and the JAK V617F positivity suggested the diagnosis of essential thrombocythemia.

Thrombocytosis may be seen in malign tumors such as renal cell and hepatocellular carcinoma, colon carcinoma.^3^ Thrombocytosis may disappear with the treatment of the tumor.^6^ It has been reported in the “sugar” clear cell tumor of the lung an increased platelet count, but, unlike our case, the platelet counts became normal after the tumor treatment.^7^ Bone marrow biopsy was performed from our patient to examine bone, but JAK mutation assessment was not performed.

Clinically, clear cell “sugar” tumor may occur in any age group (range 8 73 years) with a slight female predominance. Our case was female. These tumors are rarely associated with tuberous sclerosis and lymphangioleiomyomatosis.^8^,^9^

Teofili et al. (Blood. 2011 Mar 3;117(9):2700-7) found the Jak2 V617F mutation in endothelial progenitor cells in a subgroup of myeloproliferative neoplasms hypothesizing that this molecular alteration could be present in a common undifferentiated precursor of hematological and endothelial lineages.^10^ Since the origin of primary Clear “Sugar” Cell Tumor of the lung remains enigmatic,^2^ we hypothesized that this cell could have the Jak2 V617F mutation. Unfortunately, we were not able to perform Jak2 analysis on the tumor cells because the poor and not amplified DNA extracted by the tumor tissue.

It was detected incidentally in clinics with X-ray or thoracic CT. The patient may have hemoptysis and bloody sputum but most of them are asymptomatic.^11^ In our case, a nodule was detected by X-ray when we had been evaluated the etiology of thrombocytosis. The clinic was asymptomatic. Grossly, all of this tumors are red, gray, or brown rounded masses, varied diameter of between 1,5 to 6,5 cm, and unrelated to blood vessels or major bronchi. There was no evidence of calcification in the cases, whereas cavitations or necrosis could be observed.^8^ In our patient, the nodule’s diameter was approximately 4 cm. The diagnosis is usually more difficult with thin needle aspiration. Surgical excision is better for diagnosis and curative treatment.^7^-^9^ We preferred surgical removal for accurate diagnosis and curative treatment.

Tumor cells are usually immunoreactive with antibody for HMB-45. Although majority of these tumors are benign, rarely malign ones are also reported. There is no histological criteria reported for malignity. Infiltrative growth, increase of the cellularity, nuclear expansion, hyperchromasia, increased mitotic activity and coagulation necrosis are the prognostic factors.^8^ These findings were not observed in our case.

The differential diagnosis is extensive and includes primary tumors in the lung such as clear cell variant of bronchogenic carcinoma and acinic cell carcinoma as well as metastatic clear cell (especially renal cell carcinoma) tumors. All these tumors may present as solitary, intraparenchymal pulmonary tumors in asymptomatic patients. Owing to histological findings of the clear cell tumor, the possibility of the metastatic tumor of the kidney was evaluated in this patient. Contrary to the sugar tumor, histological examination of the renal cell carcinoma frequently shows areas of hemorrhage, necrosis, thick-walled arterial vessels, and positive fat stains;^11^,^12^ however, the histological diagnosis can be difficult; in this case the initial findings are the presence of an abdominal mass often accompanied by hematuria. Abdominal CT and renal evaluation in the 18^th^ month of the follow up was performed and did show any pathology.

In conclusion, to the best of our knowledge, this is the first case report of sugar cell lung tumor associated to essential thrombocythemia ever published. Thrombocytosis may occur especially in the formation of malign type tumor as well in lung clear cell tumor. It is important to make evaluation in terms of myeloproliferative disease in adults whose thrombocytosis continues after the elimination of primary tumor.

## Figures and Tables

**Figure 2:**
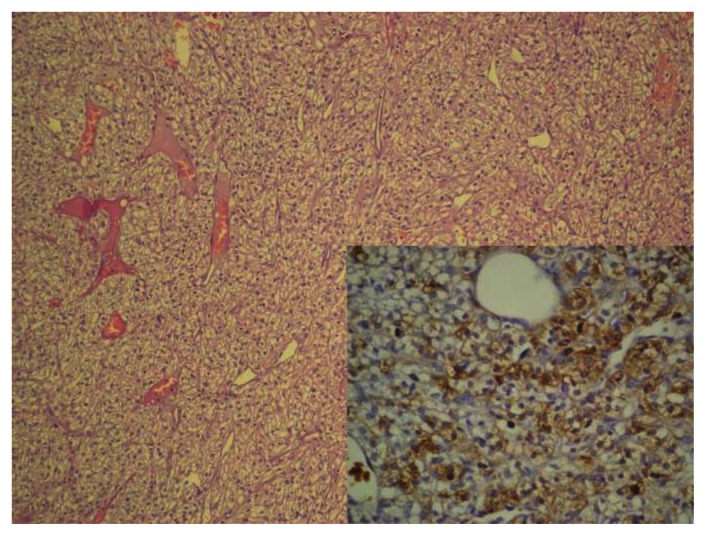
Clear cell with distinct cell borders [x100, H-E, insert: The tumor cells express HMB-45 (x400, HMB-45)]
